# Perioperative non-invasive versus semi-invasive cardiac index monitoring in patients with bariatric surgery – a prospective observational study

**DOI:** 10.1186/s12871-020-01110-x

**Published:** 2020-08-10

**Authors:** Ulf Lorenzen, Markus Pohlmann, Jonathan Hansen, Phil Klose, Matthias Gruenewald, Jochen Renner, Gunnar Elke

**Affiliations:** 1 Department of Anaesthesiology and Intensive Care Medicine, University Medical Center Schleswig-Holstein, Campus Kiel, Arnold-Heller-Str. 3 Haus R3, 24105 Kiel, Germany; 2 Department of Anesthesiology, Helios Kliniken Schwerin, 19055 Schwerin, Germany

**Keywords:** Bariatric surgery, Cardiac output, Finger-cuff, Hemodynamic monitoring, Non-invasive monitoring, Obesity, Vascular unloading technique

## Abstract

**Background:**

In morbidly obese patients undergoing laparoscopic bariatric surgery, the combination of obesity-related comorbidities, pneumoperitoneum and extreme posture changes constitutes a high risk of perioperative hemodynamic complications. Thus, an advanced hemodynamic monitoring including continuous cardiac index (CI) assessment is desirable. While invasive catheterization may bear technical difficulties, transesophageal echocardiography is contraindicated due to the surgical procedure. Evidence on the clinical reliability of alternative semi- or non-invasive cardiac monitoring devices is limited. The aim was to compare the non-invasive vascular unloading to a semi-invasive pulse contour analysis reference technique for continuous CI measurements in bariatric surgical patients.

**Methods:**

This prospective observational study included adult patients scheduled for elective, laparoscopic bariatric surgery after obtained institutional ethics approval and written informed consent. CI measurements were performed using the vascular unloading technique (Nexfin®) and semi-invasive reference method (FloTrac™). At 10 defined measurement time points, the influence of clinically indicated body posture changes, passive leg raising, fluid bolus administration and pneumoperitoneum was evaluated pre- and intraoperatively. Correlation, Bland-Altman and concordance analyses were performed.

**Results:**

Sixty patients (mean BMI 49.2 kg/m^2^) were enrolled into the study and data from 54 patients could be entered in the final analysis. Baseline CI was 3.2 ± 0.9 and 3.3 ± 0.8 l/min/m^2^, respectively. Pooled absolute CI values showed a positive correlation (r_s_ = 0.76, *P* < 0.001) and mean bias of of − 0.16 l/min/m^2^ (limits of agreement: − 1.48 to 1.15 l/min/m^2^) between the two methods. Pooled percentage error was 56.51%, missing the criteria of interchangeability (< 30%). Preoperatively, bias ranged from − 0.33 to 0.08 l/min/m^2^ with wide limits of agreement. Correlation of CI was best (r_s_ = 0.82, *P* < 0.001) and percentage error lowest (46.34%) during anesthesia and after fluid bolus administration. Intraoperatively, bias ranged from − 0.34 to − 0.03 l/min/m^2^ with wide limits of agreement. CI measurements correlated best during pneumoperitoneum and after fluid bolus administration (r_s_ = 0.77, *P* < 0.001; percentage error 35.95%). Trending ability for all 10 measurement points showed a concordance rate of 85.12%, not reaching the predefined Critchley criterion (> 92%).

**Conclusion:**

Non-invasive as compared to semi-invasive CI measurements did not reach criteria of interchangeability for monitoring absolute and trending values of CI in morbidly obese patients undergoing bariatric surgery.

**Trial registration:**

The study was registered retrospectively on June 12, 2017 with the registration number NCT03184272.

## Background

Bariatric surgery is increasingly used also in European countries as a recommended treatment option for adult patients with morbid obesity defined as a BMI ≥ 40.0 kg/m^2^ or 35.0 and 39.9 kg/m^2^ and comorbidities including type 2 diabetes or cardiorespiratory disease, respectively [1, 2]. Furthermore, patients with BMI > 30 and < 35 kg/m^2^ and type 2 diabetes may be considered for surgical treatment as well due to the beneficial effect on diabetes remission [3]. In the clinical setting of laparoscopic bariatric surgery, the combination of obesity-related physiological alterations, comorbidities, and the surgical procedure per se including the use of pneumoperitoneum (PP) and extreme changes in patient positioning contribute to an increased risk of perioperative hemodynamic complications [4, 5]. Thus, an advanced hemodynamic monitoring beyond measurement of arterial pressure is principally desirable [6]. Nowadays a number of monitoring techniques, invasive to non-invasive, continuous or intermittent, are available for cardiac index (CI) assessment such as pulmonary artery catheter, pulse-contour cardiac output (CO) monitoring or transesophageal echocardiography [6]. However, the higher the degree of invasiveness, the higher the rate of possible monitoring-associated risks such as infection, ischemia and thrombembolic events. Catheterization of a femoral or brachial arterial line for pulse-contour analysis devices may particularly bear technical difficulties due to anatomical reasons in these patients [7].

In contrast, non-invasive devices based on the vascular unloading technique first described by Penaz [8] offer the advantage of easy application and less method-immanent adverse risks [9]. These devices might be an attractive alternative for continuous advanced hemodynamic monitoring especially in this patient population. Today, the question of interchangeability of non-invasive devices compared to invasive devices is underinvestigated, particularly in the morbidly obese patient population [10]. Since no transpulmonary thermodilution CI monitoring tool has been reported to be applied intraoperatively in daily clinical routine and moreover, no recommendations are available highlighting this aspect, our department formerly decided on the basis of the available literature to use the FloTrac™ system in daily routine, when indicated [11].

Thus, the aim of our study was to compare perioperative CI measurements between the non-invasive vascular unloading technique (finger cuff method, Nexfin® system) to a semi-invasive pulse contour technique, the FloTrac™ system in a larger cohort of patients undergoing bariatric surgery. In particular, we sought to track clinical steps including PP and extreme posture changes with a likelihood of hemodynamic instability.

## Methods

### Study design and patients

This was a single-center prospective observational cohort study conducted at the Department of Anaesthesiology and Intensive Care Medicine and General Surgery, University Medical Center, Schleswig-Holstein, Campus Kiel. Inclusion criteria were defined as adult patients with an indication for elective laparoscopic bariatric surgery, a BMI ≥ 30 kg/m^2^, an ASA (American Society of Anesthesiologists) class ≥ II, and written informed consent for study participation. A BMI ≥ 30 kg/m^2^ was chosen as not only morbid obese patients defined as a BMI ≥ 40.0 kg/m^2^ or 35.0 and 39.9 kg/m^2^ and comorbidities but also obese patients with BMI > 30 and < 35 kg/m^2^ and type 2 diabetes may have been scheduled for bariatric surgery [2, 3]. Exclusion criteria were defined as aortic aneurysm > 4,5 cm, preexisting cardiac arrhythmias, peripheral arterial vascular disease Fontaine stadium > 2 and cognitive or linguistic barriers. The study protocol was approved by the local ethics committe of the Christian-Albrechts-University Kiel (file number: A 132/14) and written informed consent obtained in advance from all patients. The study was registered retrospectively on June 12, 2017 at https://clinicaltrials.gov/ct2/show/NCT03184272.

The individual risk for postoperative nausea and vomiting was evaluated at the time of study inclusion. In patients with more than 3 independent risk factors according to the score by Apfel and coworkers [12], general anesthesia was performed as total intravenous anesthesia (TIVA) using propofol and remifentanile. In the remaining patients, a balanced general anesthesia with sevoflurane or desflurane, respectively and remifentanile was used.

### Instrumentation and study protocol

The Nexfin® system (BMEYE, Amsterdam, The Netherlands, now distributed as the so-called ClearSight® system by Edwards Lifesciences, Irvine, CA, USA) is a finger cuff device combining the vascular-unloading technique with the principle of physiological calibration in order to reconstruct the brachial arterial pressure waveform from the finger arterial pressure waveform [13]. Cardiac output results from multiplication of stroke volume and heart rate where stroke volume is calculated based on the pulse contour method using the systolic blood pressure time integral. The technique has already been described in detail beforehand [14, 15]. The semi-invasive FloTrac/Vigileo™ system (Edwards Lifesciences, Irvine, CA, USA) used as the reference technique in our study only requires an arterial line and correspondingly applies an arterial pressure waveform, pulse contour analysis for stroke volume and CO calculation [16, 17].

In the run up, a non-invasive blood pressure measurement using a forearm cuff on both arms was used for all patients in order to detect physiological as well as pathological blood pressure differences. Standard clinical monitoring with non-invasive blood pressure monitoring, relaxometry and pulse oxymetrically measured oxygen saturation was performed on the right arm. In accordance to previous study protocols [18, 19], the Nexfin® and FloTrac™ system were then both connected to the ipsilateral (left) arm. Accordingly, the arterial catheter (Arrow R Intl., Reading, PA, USA; Transducer: DPT-6000, CODAN pvb Critical Care GmbH, Forstinning, Germany) was placed in the left radial artery under local anesthesia in Seldinger technique and connected to the FloTrac™ pressure transducer after checking the correct position and its patency. For the arterial pressure transducer, the zero reference point was selected at the patient’s heart height and the height was corrected accordingly to table position changes during the procedure. Initially, a zero measurement against atmospheric pressure was performed to obtain correct blood pressure values and attention was paid to an undamped pulse pressure curve.

The Nexfin® system was connected to the wrist unit as well as the heart reference system. This system adjusts the blood pressure to hydrostatic differences between the sensor and the heart level. The instruments were hold next to each other at the same level to adjust them to zero. After this procedure was completed, the heart reference system detectors were placed at finger and heart level. The correct size of the finger cuff was choosen and placed at the middle phalanx of the index finger. Finally, biometric patient data were entered as applicable in both the Vigileo™ and Nexfin® monitors and measurements of CI started.

### Data collection

**Fig. 1:**
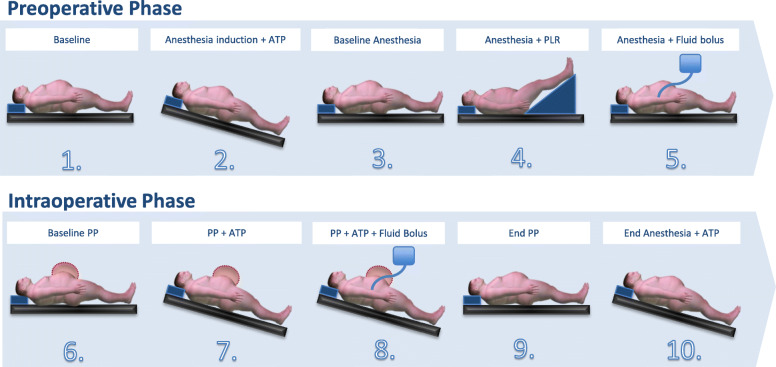
Study design and measurement time points. In the pre- and intraoperative phase cardiac index measurements with both the non- and semi-invasive monitoring devices were performed at five time points in each phase, at which hemodynamic changes were likely to occur due to clnically necessary steps of anesthesia induction, changes in posture or induction of pneumoperitoneum or fluid bolus administration. ATP: anti-Trendelenburg posture, PLR: passive leg raising, PP: pneumoperitoneum

Figure 1 illustrates the study protocol with the consecutive measurement time points of data sampling. In the preoperative and intraoperative phase, the CI was measured by both the non- and semi-invasive devices at 5 predefined measurement time points per phase at which hemodynamic changes were likely expected due to induction of general anesthesia, positioning (ATP: anti-Trendelenburg positioning, PLR: passive leg raising) and fluid bolus administration or induction of PP.

The first measurement was performed in the awake, spontaneously breathing patient in neutral position (baseline). Directly before the induction of general anesthesia, the patients were placed in ATP (30° bottom low position) as per clinical standard to improve a better oxygenation and to provide an aspiration prophylaxis (measurement time point 2). The next measurement was taken in neutral position under general anesthesia (baseline anesthesia, measurement time point 3). The following two measurement pairs (time points 4 and 5) were taken after a PLR maneuver (raising the legs by 30°) and - in neutral position - after administration of a 500 ml fluid bolus of a balanced crystalloid solution (Sterofundin® ISO, Braun, Melsungen, Germany). After this preoperative period the patients were delivered to the operating room, where the measurement tools were reconnected and a new zero balance was performed. Further measurements were taken after PP had been applied to 15 mbar (baseline PP, measurement time point 6), after the ATP positioning during surgery (measurement time point 7) and in that position after another fluid bolus of 500 ml crystalloid solution (measurement time point 8). The last two intraoperative measurements (time points 9 and 10) were carried out after termination of PP in neutral position, and again in ATP by the end of general anesthesia.

### Statistical analysis

The primary endpoint of this study was the analysis of the CI differences between the test and reference method. Sample size evaluation was based on the method by Bland and Altmann for calculating the mean measurement deviation (bias) and the precision (mean value ±1.96 standard deviation) [20, 21]. In the case of multiple measurements, the modification of the Bland-Altman method was applied (repeated measurements). The number of cases was determined with *n* = 60 patients, followed by an intermediate evaluation. For a Bland-Altman analysis, the width w of the confidence interval for the limits of agreement (LOA) is calculated as *w = 6.79 • σ • 1 / √n*, where n is number of cases and σ is the standard deviation. For a case count of *n* = 60, the result is *w = 0.88 • σ* and thus considered a sufficiently large number.

A Spearman’s correlation analysis of measurement pairs for CI between the two monitoring devices was performed followed by a Bland-Altman analysis with calculation of the mean bias and LOA defined as the standard deviation (SD) of the mean bias times 1.96 as described above [21]. In addition, the percentage error (PE) was calculated to quantify the relative differences between both measurement techniques. According to the criterion of interchangeability of a new device with the reference method, the PE must be less than 30% for CI monitoring [22]. A concordance analysis was performed to record the hemodynamic trends between the successive measurement points in the preoperative (measurement time point 1 vs. 2, 2 vs. 3, 3 vs. 4 and 4 vs. 5) and intraoperative phase (measurement time point 6 vs. 7, 7 vs. 8, 8 vs. 9 and 9 vs. 10). The measured relative (delta) changes of subsequent CI values from both devices were graphically displayed in four-quadrant plots. Pairs of measured values were excluded if - generally required by the instrument - changes in CI values of < 15% to the reference method (FloTrac™) were present. Concordance rates > 92% were accepted according to the Critchley criterion [23]. Data from descriptive analyses are listed as mean values and standard deviation or as absolute and relative frequencies where appropriate. A *P* value of < 0.05 was considered as statistically significant. Statistical analysis was performed using SPSS Statistics 21 for Windows (IBM, Armonk, NY, USA).

## Results

Sixty patients were included in the study with a mean age of 46,5 years. As the BMI was 49.2 ± 5.7 kg/m^2^, all patients met the definition for morbid obesity. Table 1 summarizes all relevant baseline patient characteristics, relevant comorbidities and type of bariatic surgery and general anesthesia performed. Table 2 shows the hemodynamic variables at each measurement time point. CI at baseline was 3.2 ± 0.9 and 3.3 ± 0.8 l/min/m^2^, respectively. Of the 10 measurement time points pre- and intraoperatively, a total of 580 pairs of measured values were computed. Six patients had to be excluded from further correlation analyses as no valid CI measurement data sets could be derived for all measurement points leaving 54 patients for the final analysis. Missing individual data points are indicated, if applicable for each of the subsequent analyses.

**Table 1:** Patient characteristics, type of bariatric surgery and general anesthesia

**Number of patients**	**60**
**Age, years**	46.5 ± 12.1
**Gender, N (%)**	female 44 (73)
	male 16 (27)
**Height, cm**	172 ± 10
**Body weight, kg**	147 ± 27
**Body mass index, kg/m**^**2**^	49.2 ± 5.7
**Comorbidities**	
Arterial hypertension, N (%)	37 (61)
Diabetes mellitus, N (%)	23 (38)
**Type of bariatric surgery**	
Gastric bypass, N	31
Sleeve gastrectomy, N	24
Gastric banding explantation, N	1
Single anastomosis duodeno-ileal bypass-sleeve gastrectomy, N	4
**Type of general anesthesia**	
Total intravenous anesthesia with propfol and remifentanile	19
Balanced anesthesia with sevoflurane and remifentanile/desflurane and remifentanile	31/10

Variables are expressed as mean ± standard deviation unless otherwise indicated in the table

**Table 2:** Hemodynamic variables at each measurement time point

Measurement time point	System	CI l/min/m^2^	SAP mmHg	DAP mmHg	MAP mmHg	HR min^− 1^
**1 Baseline**	FloTrac™	3.2 ± 0.9	153 ± 24	79 ± 12	104 ± 15	78 ± 13
	Nexfin®	3.3 ± 0.8	135 ± 23	78 ± 10	100 ± 14	
**2 Anesthesia induction + ATP**	FloTrac™	2.2 ± 0.7	103 ± 21	55 ± 12	69 ± 15	67 ± 11
	Nexfin®	2.1 ± 0.6	93 ± 19	59 ± 10	71 ± 13	
**3 Baseline Anesthesia**	FloTrac™	2.3 ± 1.1	116 ± 22	64 ± 12	79 ± 16	67 ± 12
	Nexfin®	2.3 ± 0.6	101 ± 19	61 ± 10	75 ± 14	
**4 Anesthesia + PLR**	FloTrac™	2.0 ± 0.7	117 ± 19	64 ± 10	80 ± 12	64 ± 13
	Nexfin®	2.2 ± 0.6	99 ± 17	60 ± 10	73 ± 12	
**5 Anesthesia + Fluid bolus**	FloTrac™	1.7 ± 0.7	110 ± 18	60 ± 10	74 ± 13	58 ± 10
	Nexfin®	2.0 ± 0.5	94 ± 16	57 ± 8	69 ± 11	
**6 Baseline PP**	FloTrac™	1.9 ± 1.0	119 ± 28	65 ± 14	81 ± 18	61 ± 13
	Nexfin®	2.2 ± 0.6	108 ± 23	65 ± 13	79 ± 18	
**7 PP + ATP**	FloTrac™	1.9 ± 0.6	106 ± 23	60 ± 13	74 ± 16	64 ± 11
	Nexfin®	2.1 ± 0.6	102 ± 19	65 ± 11	77 ± 14	
**8 PP + ATP + Fluid bolus**	FloTrac™	2.2 ± 0.6	119 ± 21	65 ± 12	81 ± 15	67 ± 12
	Nexfin®	2.4 ± 0.6	110 ± 19	68 ± 11	82 ± 14	
**9 End PP**	FloTrac™	2.5 ± 0.6	113 ± 19	58 ± 10	75 ± 12	65 ± 9
	Nexfin®	2.5 ± 0.5	108 ± 17	63 ± 9	79 ± 11	
**10 End Anesthesia + ATP**	FloTrac™	3.2 ± 1.0	153 ± 28	81 ± 20	104 ± 19	79 ± 17
	Nexfin®	3.2 ± 0.8	136 ± 25	79 ± 14	101 ± 20	

Variables are expressed as mean ± standard deviation

*ATP* Anti-Trendelenburg posture, *CI* Cardiac index, *DAP* Diastolic arterial pressure, *LOA* Limits of agreement, *MAP* Mean arterial pressure, *PE* Percentage error, *PLR* Passive leg raising, *PP* Pneumoperitoneum, *SAP* Systolic arterial pressure, *HR* Heart rate

### Interchangeability of all measurements

**Fig. 2:**
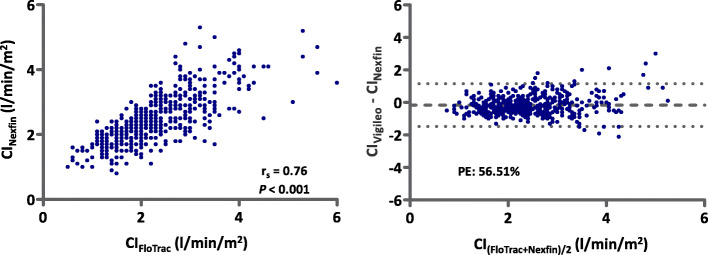
Correlation and Bland-Altman analyses of cardiac index measurements between Nexfin® and FloTrac™ for the total data sample. Pooled Spearman correlation analysis with correlation coefficient (r_s_) and *P* value shown in the left diagram. 2 data points lie outside of the axis range for better visualization of the diagram. The right diagram shows Bland-Altman plot of non-invasive (Nexfin®) and semi-invasive (FloTrac™) cardiac index (CI) measurements in l/min/m^2^. Bland-Altman analysis showing the mean difference and 95% limits of agreement for each comparison (bias ±1.96 standard deviation of the difference) as dots. 3 data points lie outside of the axis range for better visualization of the diagram. In addition, the value for the calculated percentage error (PE) is displayed in the diagram

Figure 2 shows the pooled correlation and Bland-Altman analyses of the CI measurements between Nexfin® and FloTrac™ for the total data sample. Absolute CI values recorded over all 10 measurement time points (523 value pairs) showed a positive correlation coefficient r_s_ = 0.76 (*P* < 0.001). The Bland-Altman analysis revealed a bias of − 0.16 l/min/m^2^ (LOA: − 1.48 - 1.15 l/min/m^2^). The criterion of interchangeability could not be reached with a PE of 56.51%. Table 3 summarizes correlation and Bland-Altman analysis results according to each pre- and intraoperative measurement time point. In the preoperative phase, correlation was best for measurement time point 5 after fluid bolus administration (r_s_ = 0.82, *P* < 0.001) with the lowest PE (46.34%). Bias (SD) ranged from − 0.33 (0.42) to 0.08 (0.60) l/min/m^2^ indicating a slight overestimation of the Nexfin® system at measurement time point 5 and a slight underestimation at measurement time point 2, respectively with overall wide LOA.

**Table 3:** Summary of correlation and Bland-Altman analyses according to each pre- and intraoperative measurement time point

Measurement time point	Data pairs CI_Nexfin_ vs. CI_FloTrac_ N	Correlation coeffcient r_s_	*P* value	Bias, l/min/m^2^	SD of bias, l/min/m^2^	LOA, l/min/m^2^	PE, %
**1 Baseline**	51	0.56	< 0.001	−0.08	0.90	− 1.85-1.68	54.42
**2 Anesthesia induction + ATP**	52	0.56	< 0.001	0.08	0.60	− 1.09-1.26	55.63
**3 Baseline Anesthesia**	53	0.38	0.01	−0.05	1.02	−2.06-1.96	89.64
**4 Anesthesia + PLR**	52	0.74	< 0.001	−0.20	0.50	−1.18-0.79	47.85
**5 Anesthesia + Fluid bolus**	53	0.82	< 0.001	−0.33	0.42	−1.17-0.50	46.34
**6 Baseline PP**	54	0.66	< 0.001	−0.29	0.72	−1.69-1.12	68.64
**7 PP + ATP**	52	0.64	< 0.001	−0.21	0.49	−1.16-0.75	48.88
**8 PP + ATP + Fluid bolus**	54	0.77	< 0.001	−0.18	0.42	−1.00-0.63	35.95
**9 End PP**	53	0.65	< 0.001	−0.34	0.47	−1.26-0.57	36.54
**10 End Anesthesia + ATP**	50	0.64	< 0.001	−0.03	0.78	−1.55-1.49	48.14

*ATP* Anti-Trendelenburg posture, *LOA* Limits of agreement, *PE* Percentage error, *PLR* Passive leg raising, *PP* Pneumoperitoneum, *SD* Standard deviation

### Hemodynamic trending ability of all measurements

**Fig. 3:**
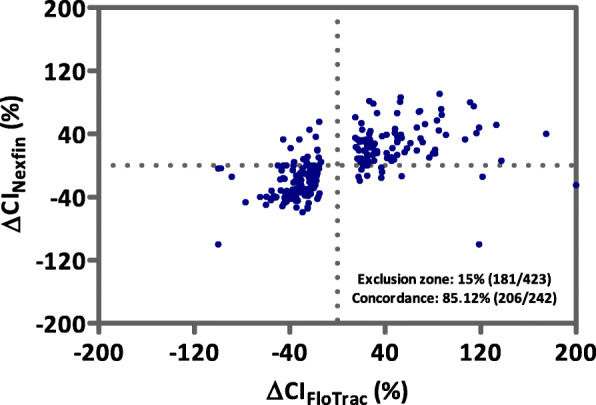
Four square plot of the concordance for cardiac index trending between Nexfin® and FloTrac™ for the total data sample. Hemodynamic trending interchangeability using a four-quadrant plot representation of the changes in cardiac index (CI) measurements from the total data sample. Data points in the left lower und right upper quadrant depict CI values with the same delta change (in %) – negative or positive. Number of values with changes in CI < 15% were excluded (exclusion zone, number of excluded values and remaining number of CI values). The concordance of the remaining values is also displayed in the diagram. An acceptable trending ability was assumed at a level of concordance > 92%. One data point lies outside the axis range

**Fig. 4:**
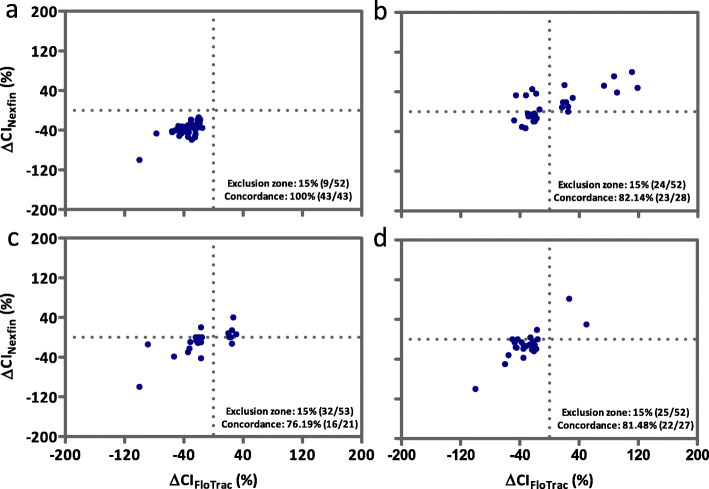
Four square plot of the concordance for cardiac index trending in the preoperative phase between Nexfin® and FloTrac™. Hemodynamic trending interchangeability using a four-quadrant plot representation of the changes in cardiac index (CI) measurements from the total data sample. Data points in the left lower und right upper quadrant depict CI values with the same delta change (in %) – negative or positive. Number of values with changes in CI < 15% were excluded (exclusion zone, number of excluded values and remaining number of CI values). The concordance of the remaining values is also displayed in the diagram. Panel **a** shows trending between baseline, patient awake and anesthesia induction with anti-Trendelenburg position, Panel **b** anesthesia induction with anti-Trendelenburg position and baseline anesthesia, Panel **c** baseline anesthesia and passive leg raising and Panel **d** anesthesia with passive leg raising and fluid bolus administration

**Fig. 5:**
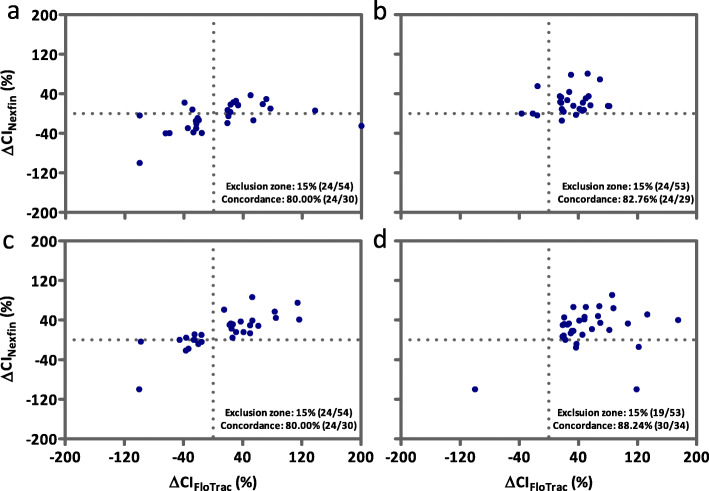
Four square plot of the concordance for cardiac index trending in the intraoperative phase between Nexfin® and FloTrac™. Hemodynamic trending interchangeability using a four-quadrant plot representation of the changes in cardiac index (CI) measurements. Data points of each diagram depict CI values in the left lower und right upper quadrant with the same delta change (in %) – negative or positive. Number of values with changes in CI < 15% were excluded (exclusion zone, number of excluded values and remaining number of CI values). The concordance of the remaining values is also displayed in the diagram. Panel **a** shows trending between anesthesia with induction of pneumoperitoneum and anti-Trendelenburg posture, Panel **b** anesthesia with pneumoperitoneum in anti-Trendelenburg posture and anesthesia with pneumoperitoneum in anti-Trendelenburg posture plus fluid bolus administration, Panel **c** anesthesia with pneumoperitoneum in anti-Trendelenburg posture plus fluid bolus administration and end of pneumoperitoneum and Panel **d** end of pneumoperitoneum and end of anesthesia in anti-Trendelenburg position

Figure 3 shows the four square plot of the concordance for CI trending between Nexfin® and FloTrac™ based measurements for the total data sample. With 41 data pairs missing and 181 valid data excluded due to the 15% exclusion zone of the reference method, 242 data pairs remained. Overall, the concordance rate of the Nexfin® system in relation to the reference method was high with 85.12% but did not reach the Critchley criterion (> 92%). Figures 4 and 5 accordingly show the four square plots of the concordance for CI trending separately for the pre- and intraoperative period.

## Discussion

In this prospective observational cohort study in 60 patients with a mean BMI of 49.2 kg/m^2^ undergoing bariatric surgery, perioperative measurements of CI by the non-invasive Nexfin® system were compared to the semi-invasive FloTrac™ device defined as the reference method at the ispsilateral arm. Overall, interchangeability for absolute CI values from 54 patients that could be entered into the final analysis, could not be shown between the two devices with a correlation coefficient of r_s_ = 0.76 (*P* < 0.001), a bias of − 0.164 l/min/m^2^ (LOA: − 1.478 - 1.150 l/min/m^2^) and a high PE (56.61%). Although a high overall concordance rate of 85.12% was found, trending capability of the vascular unloading technique in the pre- and intraoperative phase did not reach the predefined Critchley criterion of 92%.

Morbidly obese patients are prone to an increased cardiovascular risk with impaired cardiac performance, including impaired relaxation ability and myocardial hypertrophy in addition to other pre-existing comorbidities [5, 24, 25]. In the perioperative setting of (laparoscopic) bariatric surgery, hemodynamics can further be aggravated by pharmacologic agents required for general anesthesia, clinically necessary posture changes for oxygenation improvement and ease of surgical procedure, respectively as well as induction of PP [4]. In addition to heart rate and arterial blood pressure monitoring, continuous measurement of CI as the essential parameter reflecting blood flow and subsequent oxygen supply is desirable [26, 27], albeit it has yet not been shown to be related to a decrease in postoperative morbidity in this patient population. In contrast, noninvasive arterial pressure monitoring with the ClearSight® system has been reported to be associated with a significant reduction of intraoperative hypotension in two randomized trials [28, 29] and might already be sufficient to reduce postoperative morbidity when an individualized hemodynamic management is applied [30]. Modern semi-invasive (arterial cannulation) or non-invasive (finger cuff) monitoring systems are able to measure CO based on the pulse contour analysis [31, 32]. The latter systems particularly offer the advantage of avoiding technical difficulties in the morbidly obese patient and associated risks of more invasive instrumentation.

The validity of non-invasive or semi-invasive CO measurements has been demonstrated in previous studies in different populations including normal-weight patient groups and surgical settings [11, 33–35]. In morbidly obese patients undergoing bariatric surgery, 4 prospective observational studies tested the performance of the vascular unloading technique for arterial blood pressure measurements [36–39]. While the one study did not demonstrate interchangeability between the Nexfin® system and Riva-Rocci/Korotkoff-derived blood pressure measurements in 33 patients [36], the other three studies could show clinically useful trend ability of the arterial pressure values [37–39].

Thus far, evidence on non- or semi-invasive CI measurements in the perioperative setting of bariatric surgery is limited to one case series and one prospective observational study [10, 40]. In the one case series of only 8 morbidly obese patients, CO measurements by the FloTrac/Vigileo™ system were compared to the thermodilution method using a pulmonary artery catheter [40]. No valid agreement but a systematic overestimation of the semi-invasively measured CO values could be shown. This likely indicates that FloTrac™ may not be the appropriate reference technique as reported in a metaanalysis where both non- and semi-invasive technologies did not reach an acceptable level of agreement for CO monitoring in the perioperative setting [41]. In the prospective observational study including 30 patients undergoing elective bariatric laparoscopic surgery, Schraverus and coworkers compared non-invasive CO measurements by the Nexfin® system with thermodilution by the PiCCO® system as the reference [10]. No acceptable agreement between both techniques in terms of absolute values (bias of 0.60 l/min, LOA − 2.67 to 3.86 l/min, PE of 46%) and overall trend behavior (concordance rate 77%) was found. They also performed measurements at clinically relevant fixed time points including e.g. induction of anesthesia and PP. In contrast to the Schraverus study, we found a higher overall trending ability (concordance rate 85%), with a 100% concordance rate found between baseline and induction of anesthesia in ATP posture (measurement time points 1 and 2). At all other measurement time points in the pre- and intraoperative phase, concordance rates of trending CI were between 76 and 88%. Albeit the predefined Critchley criterium was also not reached in our study, an overall concordance rate of 85% otherwise allows to analyze delta values less than 10–15%, representing 42% of the data pairs excluded as predefined. Thus, using the non-invasively derived preoperative trending behavior as information on the presence or absence of CI variation likely offers the possibility to render a different and personalized intraoperative hemodynamic treatment, e.g. the decision for fluid administration [42]. Taking into account that both systems calculate the CI as a cardiac flow marker from the blood pressure curve, non- to moderately valid blood pressure values can already inevitably lead to greater inaccuracy in the determination of CI. This already applies to stable hemodynamic situations as recently demonstrated in a meta-analysis of 28 studies with a total of 919 patients [43]. Precision may further be impaired by hemodynamic instability as particularly studied here at the different mesasurement time points including induction of general anesthesia, posture changes, fluid administration and PP. Some authors assume interchangeability with the gold standard only during stable hemodynamics [19]. The majority of comparative studies for cardiac flow markers originate from the field of cardiac surgery with good agreement found in comparison to the pulmonary artery catheter [33] or the PiCCO system [35]. However, it was pointed out that the results are not necessarily transferable to other patient cohorts and devices or techniques used for CI measurement, respectively. In another meta-analysis of 20 studies in 624 pediatric patients, accuracy and precision of different non- and semi-invasive devices and (invasive) reference methods for CO monitoring in pediatric patients showed that the overall pooled bias and PE were 0.13 ± 0.44 l/min (95% LOA: − 0.74 to 0.99 l/min) and 29.1%, respectively [44]. Although the bias was small, the pooled PE was around the acceptable limit of 30%. In a subgroup analysis by the type of device, the pooled mean bias and PE were 0.32 ± 0.64 l/min and 33.0% for pulse contour analysis with still a high heterogeneity accounting for device type.

The two measurement systems analysed in our study estimate individual vascular compliance by computer-assisted databases using the body weight besides other biometric data including sex, age and height [14–16]. In a recent study on 30 patients (BMI ≥ 35 kg/m^2^) undergoing gastric bypass surgery, Boly et al. investigated whether different body weight formulas play a role in possible differences of CO measurements using Nexfin® as compared to invasive thermodilution (PiCCO® system) [45]. Using adapted body weight (calculated by ideal body weight + 0.4 [actual - ideal body weight]) for the calibration of both devices thereby showed the best agreement of CO values as compared to actual body weight or ideal body weight (calculated by the formula: 22 × length (m)^2^). However, the evaluation of the CI (ratio of CO to body surface area) in our study most likely minimized the possible influence of body weight as one of the underlying calibration variables. Moreover, body weight per se is not the only factor to adequately reflect individual vascular compliance. Especially in the morbidly obese patient cohort arterial compliance can be altered in the presence of obesity-associated comorbidities [46]. The CO-Trek analysis used by the Nexfin® system is based on the “Modelflow” method, which simulates a three-element air vessel system including aortic impedance, compliance and peripheral resistance [33]. Thus, the wider LOA between the Nexfin® system and the FloTrac™ reference method detected in our study may rather be explained by insufficient calculations of the input impedance by the Nexfin® algorithm ultimately resulting in the oberserved under- or overestimation of absolute CI values at the respective measurement time points.

With regard to preexisting vasculopathies likely invalidating measurements with the finger-cuff method used, the presence of peripheral arterial occlusive disease or advanced secondary damage from diabetes mellitus or arterial hypertension encompassed in the metabolic syndrome also play a role as comorbidities in the investigated patient population [47]. However, these vasculopathic confounding factors were rather unlikely in our study since a higher degree of arterial occlusive disease was defined as an exclusion criterion. The patients were relatively young at 46.5 years and only 38% were diagnosed with preexisting diabetes mellitus while arterial hypertension was present in two thirds of the patients. We did not systematically analyse differences between patients with and without those preexisting comorbidities, however, no systematic methodological problems with the finger cuff derived measurement signals, e.g. due to anatomical reasons became evident at baseline.

Besides the observational nature of our study, the main limitation is that we did not use an invasive gold standard, i.e. thermodilution method as a reference for CO determination. In consideration of the high invasiveness and associated risk in particular with pulmonary artery catheters the risk-benefit ratio was considered to be not justified [6]. Since no transpulmonary thermodilution CI monitoring tool is essentially recommended to be applied intraoperatively in daily clinical routine, particularly for the patient population studied, our department formerly decided on the basis of the available literature to use the FloTrac™ system in daily routine, when indicated [11]. Thus, we deliberately chose the semi-invasive pulse-contour analysis method as a reference. Alternative semi-invasive devices for non-calibrated pulse-contour analysis-based CO measurement are currently under investigation in the context of perioperative, individualized hemodynamic optimization in a large, international randomized controlled study [48]. A further limitation may be that the ipsilateral measurement of intra-arterial pressure from the reference radial line likely introduced bias for the finger cuff photopletysmography based measurements of the small finger arteries. Kurki et al. were able to show that cannulation of the radial artery generally reduces the blood flow distal to the puncture site although to varying degrees between individuals [49]. We did not measure arterial pressure on both upper arms at baseline in order to identify potential pressure differences between the left and right arm as conducted in the study of Rogge et al. [38]. However, contralateral measurement may have also introduced bias due to differences in vessel architecture. Moreover, patients were excluded from study participation if they presented with peripheral vascular disease (Fontaine stadium ≥2) or arrhythmias and as mentioned before less than half of our patients had diabetes.

A strength of our study is that it provides – to our best knowledge - the largest sample size of morbidly obese patients with continuous CI measurements during bariatric surgery to date. Furthermore, this study thoroughly reflected the clinical scenarios patients undergo in the perioperative setting including different postures, induction of anesthesia and fluid bolus administration as well as on- and off-set of PP.

In conclusion, non-invasive CI measurements using the vascular unloading technique based Nexfin® system in the perioperative phase of morbidly obese patients was not interchangeable with the semi-invasive reference method, neither with respect to absolute nor relative (trending) values. However, we have observed a strong trending ability in non-invasive CI measurements during the sensitive period of induction of anesthesia. Our findings underline further demand of larger trials to better evaluate the clinical useability of non- and semi-invasive devices for continuous CI measurements in the growing field of bariatric perioperative medicine.

## Data Availability

The datasets used and/or analyzed during the presented study are available in an anonymous fashion from the corresponding author on reasonable request.

## Abbreviations

ATP: Anti-Trendelenburg posture
BMI: Body mass index
CI: Cardiac index
CO: Cardiac output
DAP: Diastolic arterial pressure
HR: Heart rate
LOA: Limits of agreement
MAP: Mean arterial pressure
PE: Percentage error
PLR: Passive leg raising
PP: Pneumoperitoneum
SAP: Systolic arterial pressure
SD: Standard deviation
TEE: Transesophageal echocardiography
TIVA: Total intravenous anesthesia
w: Width
